# Maternal obesity and postpartum haemorrhage after vaginal and caesarean delivery among nulliparous women at term: a retrospective cohort study

**DOI:** 10.1186/1471-2393-12-112

**Published:** 2012-10-18

**Authors:** Elaine M Fyfe, John MD Thompson, Ngaire H Anderson, Katie M Groom, Lesley M McCowan

**Affiliations:** 1 Department of Obstetrics and Gynaecology, University of Auckland, Private Bag, 92019, Auckland, New Zealand; 2 Department of Paediatrics, University of Auckland, Private Bag, 92019, Auckland, New Zealand

**Keywords:** Nulliparity obesity postpartum haemorrhage

## Abstract

**Background:**

Increasing rates of postpartum haemorrhage in developed countries over the past two decades are not explained by corresponding changes in risk factors and conjecture has been raised that maternal obesity may be responsible. Few studies investigating risk factors for PPH have included BMI or investigated PPH risk among nulliparous women. The aim of this study was to determine in a cohort of nulliparous women delivering at term whether overweight and obesity are independent risk factors for major postpartum haemorrhage (PPH ≥1000ml) after vaginal and caesarean section delivery.

**Methods:**

The study population was nulliparous singleton pregnancies delivered at term at National Women’s Hospital, Auckland, New Zealand from 2006 to 2009 (N=11,363). Multivariable logistic regression was adjusted for risk factors for major PPH.

**Results:**

There were 7238 (63.7%) women of normal BMI, 2631 (23.2%) overweight and 1494 (13.1%) obese. Overall, PPH rates were increased in overweight and obese compared with normal-weight women (n=255 [9.7%], n=233 [15.6%]), n=524 [7.2%], *p* <.001) respectively. There was an approximate twofold increase in risk in obese nulliparous women that was independent of confounders, adjusted odds ratio [aOR (95% CI)] for all deliveries 1.86 (1.51-2.28). Being obese was a risk factor for major PPH following both caesarean 1.73 (1.32-2.28) and vaginal delivery 2.11 (1.54-2.89) and the latter risk was similar after exclusion of women with major perineal trauma and retained placentae. Three additional factors were consistently associated with risk for major PPH regardless of mode of delivery: increasing infant birthweight, antepartum haemorrhage and Asian ethnicity.

**Conclusion:**

Nulliparous obese women have a twofold increase in risk of major PPH compared to women with normal BMI regardless of mode of delivery. Higher rates of PPH among obese women are not attributable to their higher rates of caesarean delivery. Obesity is an important high risk factor for PPH, and the risk following vaginal delivery is emphasised. We recommend in addition to standard practice of active management of third stage of labour, there should be increased vigilance and preparation for PPH management in obese women.

## Background

The incidence of postpartum haemorrhage (PPH) has been increasing in several developed countries over the past two decades, with rates rising by over one third [1-3]. This disturbing rise, with its associated maternal morbidity and mortality, [4] is not explained by corresponding changes in risk factors such as increased rates of caesarean section and induction of labour [5,6]. A contemporaneous rise in global obesity has raised conjecture that maternal obesity may be responsible for this increase in PPH rates [3]. Associations between obesity and PPH have been reported in several studies investigating the relationship between increased BMI and birth outcomes in a general obstetric population [7,8]. Studies investigating specific risk factors for PPH have demonstrated that nulliparous women have elevated rates of PPH compared to those who are multiparous [9-11]. Nulliparous women comprise a large sub-group of the birthing population especially in Western countries. Amongst studies specifically investigating a variety of risk factors for PPH, maternal BMI is rarely considered as a potential risk factor. In these few studies, results are inconsistent with one showing no association between BMI and PPH [11], but others showing a positive association [12,13]. One study has directly investigated maternal BMI and risk of PPH, and in this group of women of mixed parity, increasing BMI was associated with increased risk of PPH [14].

Maternal obesity is associated with an elevated risk of intrapartum caesarean section, predominantly for failure to progress, [15-17] the mechanism of which is suggested to be due to reduced uterine contractility [17]. Among nulliparous women in labour at term, we have previously shown that the elevated risk of intrapartum caesarean is confined to the first stage of labour, as obese women who progress to the second stage of labour are just as likely to deliver vaginally as women with normal BMI [16]. We speculate therefore that obese women who give birth vaginally may have normal myometrial contractility. Uterine atony, the leading cause of PPH, has been associated with slow progress in labour, a surrogate for impaired intrapartum myometrial contractility [11]. This has led us to hypothesise in this retrospective cohort study of nulliparous women who delivered at term, that 1. overweight and obese women who delivered vaginally would not have increased rates of major PPH (≥1000mls) compared to women with normal BMI, and 2. overweight and obesity would be independent risk factors for major PPH (≥1000mls) in women who had a caesarean section.

## Methods

**Figure 1:**
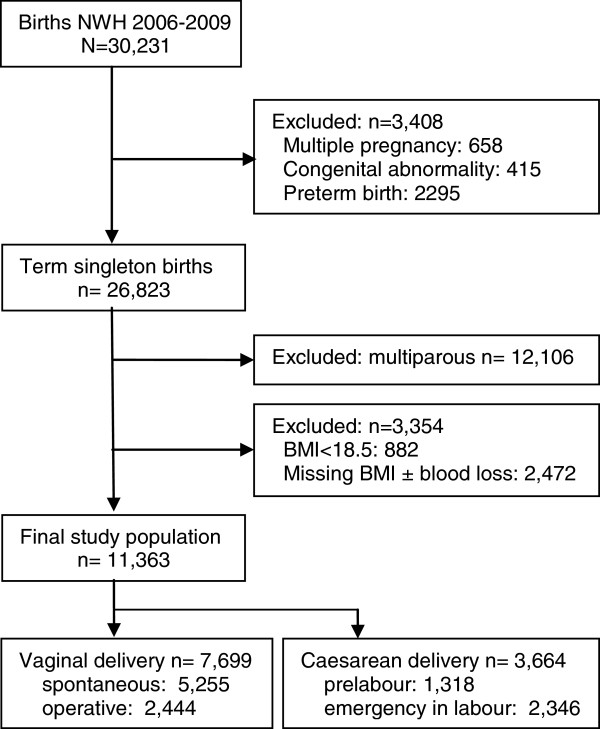
Recruitment flow chart.

The National Women’s Health (NWH) clinical database of births from Jan 2006 to Dec 2009 was used for this retrospective cohort study. NWH is a tertiary referral hospital in Auckland, New Zealand with a diverse ethnic population and approximately 7500 deliveries per year. The NWH database of births consists of de-identified, prospectively collected maternity data for all births occurring at greater than or equal to 20 weeks of gestation, which includes demographic data, antenatal complications, and detailed delivery and newborn data. Data are routinely checked for completeness, out of range values and inconsistency [18]. Ethical approval for this study was gained from the Northern X Regional Ethics Committee (NTX/09/179/EXP). The final study population included nulliparous women with a singleton pregnancy who delivered a live infant at term (Figure 1).

Maternal body mass index (BMI) (kg/m^2^) was calculated using maternal height and weight measured to the nearest centimetre and kilogram respectively at the first antenatal booking visit, and was available for 91.2% of the term study population. As the focus of the current study was overweight and obesity, underweight women (BMI less than 18.5), who comprised a small proportion of this population (6.0%), were excluded. Women were classified into normal, overweight and obese groups according to conventional World Health Organization (WHO) BMI criteria: normal (18.5-24.9 kg/m^2^), overweight (25–29.9 kg/m^2^), and obese (≥30 kg/m^2^) [19]. Normal BMI was the referent group. The primary outcome measure was major primary postpartum haemorrhage defined as blood loss equal to or greater than 1000mls [20] within 24hrs of delivery as recorded on the delivery summary by the attending midwife or doctor. Standard practice for estimation of blood loss is a combination of visualisation and measurement by weight. This clinically relevant amount was selected as a lesser blood loss of between 500mls and 1000mls is not uncommon and is not associated with adverse outcomes in the majority of healthy women [21,22]. Active management of the third stage of labour, 10IU of intramuscular oxytocin at the time of delivery of the anterior shoulder, is recommended standard practice within our unit. Estimated date of delivery (EDD) was calculated from a certain last menstrual period date or by ultrasound scan as per the Australasian Society for Ultrasound in Medicine guidelines [23,24]. Ethnicity was self-determined and prioritised as per New Zealand Ministry of Health guidelines [25]. Antepartum haemorrhage (APH) was defined as vaginal bleeding from any cause at or beyond 20 weeks of gestation after excluding placenta praevia [18]. Gestational hypertension and preeclampsia were defined as per International Society for the Study of Hypertension in Pregnancy criteria [26]. Gestational Diabetes Mellitus (GDM) was defined in accordance with The Australian Diabetes in Pregnancy Society [27].

Term delivery was delivery at a gestation of 37 weeks and 0 days or greater. Active labour was defined as regular, painful uterine contractions with progressive cervical effacement and dilation and cervical dilatation ≥3cm [28]. Prelabour elective caesarean section delivery was a planned procedure before or following the onset of labour, when the decision for caesarean section was made before labour [29]. Prelabour emergency caesarean section delivery was a delivery required because of an emergency situation (e.g. fetal distress) before the onset of active labour when the caesarean section was performed having not been previously considered necessary. Emergency caesarean section in labour was delivery required because of an emergency situation in active labour (e.g. failure to progress, obstructed labour, fetal distress) when the caesarean section was performed having not been previously considered necessary [29]. Perineal tears were defined as per Royal College of Obstetricians and Gynaecologists [30]. Retained placenta was failure of placental delivery within 60 minutes after delivery of the fetus [31]. Small for gestational age (SGA) and large for gestational age (LGA) were defined as infant birthweight <10^th^ and >90^th^ customised centile respectively [32].

Univariable logistic regression was performed to compare maternal characteristics and antenatal, birth and neonatal outcomes for women who had major PPH compared with those who did not. This analysis was undertaken for all births and then repeated for vaginal and caesarean deliveries separately. Multivariable logistic regression for major PPH was performed adjusting for potential confounders identified after an extensive literature review. The following variables of hypothesised interest or potential confounders were hence included in the model: BMI, maternal age; ethnicity; smoking; maternity care provider; antepartum haemorrhage; diabetes; hypertension; induction of labour; epidural anesthesia; duration of first, second and third stages of labour; mode of delivery; perineal trauma; retained placenta and birthweight [1,8,14,33,34]. As all of the characteristics and outcomes in univariable analyses were potential confounders, all covariates were included in the multivariable models. Data analyses were performed using SAS^©^ version 9.2. (SAS Institute Inc., Cary, NC).

## Results

**Figure 2:**
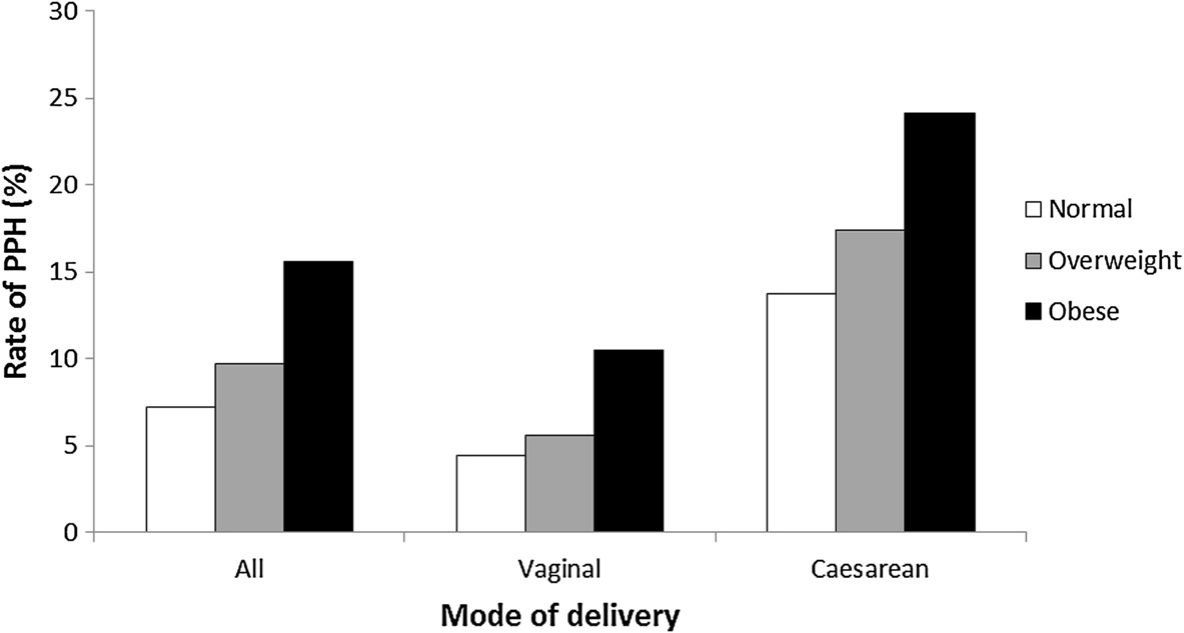
Rate of major PPH (≥1000mls) by maternal BMI according to mode of delivery.

Of 30,231 births at National Women’s Hospital, Auckland, between January 2006 and December 2009, 12,407 (41%) were in nulliparous women, of whom 11,363 (92%) met the entry criteria for this study. Vaginal delivery occurred in 7699 (67.8%), and caesarean section in 3664 (32.2%) women (Figure 1). Prevalence of major postpartum haemorrhage (PPH) overall was 8.9%, [vaginal delivery (5.4%); caesarean section (16.2%)]. Among the whole population, being overweight or obese was associated with an increased risk for PPH (OR 1.38, 95% CI 1.18-1.61 and 2.37, 95% CI 2.01-2.79 respectively). Other risk factors for PPH among all deliveries in univariable analysis were Pacific Island or Asian ethnicity, a history of antepartum haemorrhage (APH) or hypertensive disorders, induction of labour and retained placenta (Table 1). Compared to spontaneous vaginal delivery, forceps and caesarean section were associated with a two to threefold increase in risk of major PPH respectively. Increasing infant birthweight also increased risk of major PPH. After adjustment for confounding factors, there was a dose dependent relationship between BMI and risk of major PPH, which was more common among women who were overweight 255 (9.7%) or obese 233 (15.6%) compared with those with normal BMI 524 (7.2%), (aOR 1.20, 95% CI 1.01-1.42 and 1.86, 95% CI 1.51-2.28 respectively) (Figure 2). Other results were similar following adjustment for confounding factors.

**Table 1:** Risk and protective factors for major postpartum haemorrhage (≥1000mls) for term nulliparous women – all deliveries

	**No PPH**	**PPH**		
	**n= 10351 (91.1%)**	**n= 1012 (8.9%)**	**Unadjusted OR’s**	Adjusted OR’s*
Maternal characteristic				
BMI^†^				
18.5-24.9	6714 (64.9)	524 (51.8)	1.00	1.00
25.0-29.9	2376 (22.9)	255 (25.2)	1.38 (1.18-1.61)	**1.20 (1.01-1.42)**
≥ 30.0	1261 (12.2)	233 (23.0)	2.37 (2.01-2.79)	**1.86 (1.51-2.28)**
Age (y)				
Less than 20	551 (5.3)	38 (3.8)	0.70 (0.49-0.98)	0.77 (0.53-1.12)
20-29	4078 (39.4)	405 (40.0)	1.00	1.00
30-34	3553 (34.3)	336 (33.2)	0.95 (0.82-1.11)	0.94 (0.79-1.11)
≥ 35	2169 (21.0)	233 (23.0)	1.08 (0.91-1.28)	0.93 (0.77-1.14)
Ethnicity				
European	5573 (53.9)	491 (48.5)	1.00	1.00
Maori	765 (7.4)	78 (7.7)	1.16 (0.90-1.49)	1.27 (0.95-1.69)
Pacific island	881 (8.5)	136 (13.5)	1.75 (1.43-2.15)	**1.60 (1.25-2.06)**
Asian	2052 (19.8)	227 (22.4)	1.26 (1.06-1.48)	**1.61 (1.34-1.94)**
Indian	766 (7.4)	64 (6.3)	0.95 (0.72-1.24)	1.20 (0.89-1.61)
Other	314 (3.0)	16 (1.6)	0.58 (0.35-0.96)	0.68 (0.40-1.15)
Smoking (at booking)				
No	9086 (87.8)	908 (89.7)	1.00	1.00
Yes	778 (7.5)	69 (6.8)	0.89 (0.69-1.15)	0.86 (0.65-1.14)
Unknown	487 (4.7)	35 (3.5)	0.72 (0.51-1.02)	**0.67 (0.47-0.97)**
Maternity care				
Private	2556 (24.7)	231 (22.8)	1.00	1.00
Public	7795 (75.3)	781 (77.2)	1.11 (0.95-1.29)	**0.83 (0.69-0.99)**
Pregnancy complications				
Antepartum haemorrhage^‡^				
No	9899 (95.6)	924 (91.3)	1.00	1.00
Yes‡	378 (3.7)	60 (5.9)	1.70 (1.28-2.25)	**1.76 (1.31-2.36)**
Placenta praevia	74 (0.7)	28 (2.8)	4.06 (2.61-6.30)	**4.00 (2.48-6.47)**
Diabetes				
Nil	7651 (73.9)	733 (72.4)	1.00	1.00
Type 1 or Type 2	64 (0.6)	8 (0.8)	1.31 (0.62-2.73)	0.82 (0.38-1.80)
Gestational	463 (4.5)	52 (5.2)	1.17 (0.87-1.58)	0.92 (0.66-1.29)
Unknown	2173 (21.0)	219 (21.6)	1.05 (0.90-1.23)	1.06 (0.90-1.26)
Hypertension				
Nil	9332 (90.2)	878 (86.8)	1.00	1.00
Chronic	143 (1.4)	26 (2.6)	1.93 (1.27-2.95)	**1.78 (1.13-2.80)**
Gestational	523 (5.0)	56 (5.5)	1.14 (0.86-1.51)	0.98 (0.72-1.33)
Preeclampsia	353 (3.4)	52 (5.1)	1.57 (1.16-2.11)	1.33 (0.95-1.85)
Induction of labour	3187 (30.8)	397 (39.2)	1.45 (1.27-1.66)	**1.20 (1.03-1.40)**
Delivery outcomes				
Mode of delivery				
Vaginal				
Spontaneous	5010 (48.4)	245 (24.2)	1.00	1.00
Ventouse	1561 (15.1)	102 (10.1)	1.34 (1.05-1.70)	**1.40 (1.09-1.79)**
Forceps	711 (6.9)	70 (6.9)	2.01 (1.53-2.66)	**1.98 (1.49-2.65)**
Caesarean				
Prelabour	1165 (11.2)	153 (15.1)	2.69 (2.17-3.32)	**2.99 (2.33-3.83)**
Emergency in labour	1904 (18.4)	442 (43.7)	4.75 (4.03-5.60)	**4.47 (3.74-5.35)**
Retained placenta	109 (1.1)	38 (3.8)	3.67 (2.52-5.33)	**6.60 (4.42-9.85)**
Neonatal outcomes				
Gestation at delivery (days)	278.7 ± 8.6	279.8 ± 8.6	1.11 (1.05-1.17) per ↑1wk	0.96 (0.90-1.03) per ↑1wk
Birthweight (g)	3416 ± 462	3621 ± 511	1.58 (1.47-1.69) per ↑500g	**1.49 (1.38-1.62) per ↑500g**
Small for gestational age	1205 (11.6)	84 (8.3)	0.69 (0.55-0.87)	§
Large for gestational age	848 (8.2)	178 (17.6)	2.39 (2.01-2.85)	§

Data are n (%) or mean ± standard deviation.

Significant adjusted OR’s are bolded.

* Adjusted for all variables in table.

^†^Body Mass Index.

^‡^from any cause excluding placenta praevia >20wk gestation.

§ SGA/LGA not included in multivariable model.

Among women who delivered vaginally, independent risk factors were similar to those for the whole population (Table 2). Higher BMI was associated with an increased risk of major PPH (normal BMI 223 [4.4%], overweight 96 [5.6%], and obese 98 [10.5%]). After adjustment for covariates, obese women had a twofold increase for major PPH compared to women with normal BMI [aOR 2.11, 95% CI 1.54-2.89]. Other independent risk factors for major PPH in women with vaginal birth are shown in Table 2. In subgroup analyses, women with risk factors associated with non-atonic PPH, namely episiotomy, third/ fourth degree perineal lacerations (n=230) or retained placenta (n=147) were excluded. The resulting independent risk factors were the same with a similar magnitude of effect (data not shown), demonstrating that the association between obesity and PPH was not attributable to perineal trauma or retained placenta.

**Table 2:** Independent risk factors for major postpartum haemorrhage (≥1000mls) after vaginal delivery in term nulliparous women

**Risk factor**	Adjusted OR’s*
BMI ≥ 30.0	2.11 (1.54-2.89)
Ethnicity	
Pacific island	2.30 (1.62-3.27)
Asian	1.64 (1.22-2.20)
Antepartum haemorrhage^†^	1.91 (1.25-2.91)
Pre-eclampsia	1.97 (1.22-3.19)
Induction of labour	1.46 (1.15-1.86)
Third stage of labour > 15min^‡^	1.62 (1.18-2.22)
Retained placenta	4.88 (3.08-7.73)
Episiotomy	1.94 (1.19-3.19)
3^rd^ or 4^th^ degree tear	5.09 (2.91-8.89)
Birthweight (g) ↑ 500g	1.39 (1.22-1.59)

Data are adjusted odds ratio (95% confidence interval).

* Adjusted for all variables in Table 1 plus duration of 1^st^, 2^nd^ and 3^rd^ stages of labour and perineal trauma (including graze, second degree tear).

^†^from any cause excluding placenta praevia >20wk gestation.

^‡^ compared with third stage 5–10 minutes.

Emergency caesarean section in labour was associated with a higher risk for major PPH compared with prelabour caesarean section (aOR 4.47, 95% CI 3.74-5.35 versus aOR 2.99, 95% CI 2.33-3.83) (Table 1). However, there was no significant difference in magnitude of effect related to any of the risk factors for major PPH between prelabour and emergency intrapartum caesarean section (data not shown). This demonstrated that the increase in risk with emergency intrapartum caesarean was not explained by any of the variables included in this study. Therefore, all caesarean sections were combined and analysed as a single entity (Table 3). Among women with caesarean sections, those who were obese had a near twofold increase in the rate of major PPH compared to women with normal BMI (normal [13.7%], overweight [17.4%], obese [24.2%]) (Figure 2). This increase in risk associated with obesity was very similar following adjustment for covariates (aOR 1.73, 95% CI 1.32-2.28), consistent with our findings after vaginal delivery (Table 3). Other independent risk factors for major PPH after caesarean section are shown in Table 3. Obesity was one of four factors (along with Asian ethnicity, antepartum haemorrhage and increasing infant birthweight) consistently associated with risk for major PPH regardless of mode of delivery. When we investigated the relationship between birthweight by z-scores (thus accounting for gestational age) and major PPH, we demonstrated a linear relationship between birthweight z-scores and adjusted odds ratios of major PPH (data not shown).

**Table 3:** Independent risk factors for major postpartum haemorrhage (≥1000mls) after caesarean delivery in term nulliparous women

**Risk factor**	Adjusted OR’s*
BMI ≥30.0	1.73 (1.32-2.28)
Asian ethnicity	1.57 (1.23-2.01)
Public obstetric care	1.39 (1.11-1.74)
Antepartum haemorrhage^†^	1.65 (1.08-2.52)
Placenta praevia	3.08 (1.91-4.98)
Chronic hypertension	1.90 (1.08-3.35)
Birthweight (g) ↑ 500g	1.48 (1.34-1.64)

Data are adjusted odds ratio (95% confidence interval).

* Adjusted for all variables in Table 1.

^†^from any cause excluding placenta praevia >20wk gestation.

## Discussion

We have demonstrated that obese nulliparous women delivering a singleton infant at term have a twofold increase in risk of major PPH, regardless of mode of delivery, and that this risk is independent of many other recognised risk factors for major PPH. Contrary to our hypothesis we have found that obese nulliparous women who give birth vaginally have a twofold increase in risk of major PPH, similar in magnitude to those delivering by caesarean section. Previous studies have suggested that the increase in risk of PPH in obese women was largely explained by a concurrent increased caesarean section rate [35,36]. Our findings identifying elevated risk for major PPH in obese women after vaginal birth are an important alert for clinicians. Active management of third stage is already recommended as standard practice for all women [37]. We would recommend in this group of women who are obese, additional vigilance is required to prevent and manage PPH. No other studies have primarily investigated the role of maternal obesity on risk of major PPH among nulliparous women. The rate of major PPH (greater than or equal to1000mls) in this nulliparous cohort was 8.9%, (vaginal delivery [5.4%]; caesarean section [16.2%]). This is higher than the rate reported in a previous study in women of mixed parity (5.3%) where a higher threshold was used for major PPH, namely blood loss greater than 1000mls [14]. If this same definition is applied to our population (i.e. >1000mls), the rates of major PPH are almost identical (5.1% in our cohort). Among the few previous publications reporting maternal obesity and general birth outcomes in nulliparous women, comparisons are difficult due to absent or differing definitions of PPH and none have assessed the risk of PPH after vaginal delivery and caesarean section separately. Our findings are consistent with the only study of nulliparous women that adjusted for confounding factors, and reported a two fold increase in risk of major PPH in obese women [35]. However, in that study vaginal and caesarean births were not analysed separately, and no adjustment was made for perineal trauma or birthweight which are consistently reported as risk factors for PPH [34]. Two other studies in nulliparous women have reported no association between obesity and major PPH but these studies were underpowered [38,39]. In a further study, increased rates of PPH (blood loss greater than 500mls) were reported after vaginal birth in nulliparous obese women compared to those with a BMI of 20–30, but the magnitude of risk was not quantified [40].

Only one other study has primarily investigated the relationship between maternal obesity and PPH (defined as haemorrhage >1000mls) [14]. Blomberg reported a small increased risk among obese women following vaginal delivery, but PPH risk was variable according to class of obesity following caesarean section. This study population was of mixed parity, and adjustment was only possible for very limited confounders (year of infant birth, maternal age, parity and smoking). Blomberg reported an increased rate of PPH in obese women predominantly associated with uterine atony, but also due to soft tissue trauma. No association was reported between obesity and PPH due to retained placenta. Although we did not have data available to identify the primary cause of PPH, when we performed a subgroup analysis amongst women who delivered vaginally excluding those with major perineal trauma (episiotomy or third/fourth degree laceration), and/or retained placenta, there was no significant difference in effect. We therefore demonstrated that the higher rates of PPH in obese women in our study were not attributable to either major perineal trauma or retained placenta and hence were most likely due to uterine atony. Other risk factors we found for major PPH after vaginal birth were similar to those previously identified, as seen in Table 2.

It is well established that obese women have higher rates of caesarean section, especially emergency intrapartum caesarean section, [16] and we confirmed that this mode of delivery was associated with the highest rates of PPH among the whole study population. We found that obese women who had a caesarean section had a 70% increase in risk for PPH. Challenging surgery in obese patients is associated with prolonged operative time, and consequently with increased blood loss [41]. Other risk factors that we identified for PPH after caesarean section are consistent with previous studies [5,33,41-43].

Obesity was one of four factors we identified that independently increased risk for PPH after both vaginal and caesarean deliveries among nulliparous women (along with increasing birthweight, APH and Asian ethnicity). The relevance of our finding that increasing birthweight is associated with risk of PPH is that we have shown that the relationship is dose dependent and that there is not a cut off at which increased risk occurs, for example with macrosomic or large for gestational age infants, which has been reported previously [44,45]. Contextualising this finding to the clinical setting, there is a 40% increase in risk for PPH with every 500g increase in birthweight in term infants.

Our novel finding that the independent risk for PPH was increased in women with a history of APH from any cause exclusive of placenta praevia is clinically significant as APH (predominantly of unknown origin) occurred in 5.2% of our nulliparous cohort. An association between PPH and Asian ethnicity has been reported previously [34,45,46].

A strength of our study was the ability to adjust for many known risk factors for PPH to better determine the independent effect of obesity for vaginal and caesarean delivery discretely. In keeping with other clinical studies, visual estimation was usual practice to estimate blood loss in our study [34]. Visual estimation has been reported to underestimate higher volumes of blood loss, [47] it is therefore unlikely that we overestimated major PPH prevalence. We did not have data to further assess PPH defined by peripartum reduction in haemoglobin or requirement for blood transfusion.

## Conclusions

We advocate the inclusion of obesity in future research investigating risk factors for major PPH as recommended by the International Postpartum Hemorrhage Collaborative Group [3]. Nulliparous obese women should be regarded as high risk for PPH, with a twofold increase in risk of major PPH (≥1000mls) compared to normal weight women regardless of mode of delivery. Therefore we recommend in addition to standard practice of active management of third stage of labour, there should be increased vigilance and preparation for PPH management in obese women.

### Details of ethics approval

Ethical approval for this study was gained from the Northern X Regional Ethics Committee (NTX/09/179/EXP).

## Competing interests

The authors declare that they have no competing interests.

## Authors’ contributions

EF conceived of the study and participated in its design, performed the statistical analysis, interpreted the data and drafted and revised the manuscript. LMcC conceived of the study and participated in its design, interpreted the data and helped to draft and revise the manuscript. JT performed the statistical analysis, interpreted the data, and helped to draft and revise the manuscript. NA interpreted the data and helped to draft and revise the manuscript. KG interpreted the data and helped to draft and revise the manuscript. All authors read and approved the final manuscript.

## Pre-publication history

The pre-publication history for this paper can be accessed here:

http://www.biomedcentral.com/1471-2393/12/112/prepub
